# Delayed pancreatic metastasis from renal cell carcinoma managed with pazopanib and stereotactic ablative body radiotherapy

**DOI:** 10.1093/jscr/rjaf798

**Published:** 2025-10-06

**Authors:** Mutahar A Tunio, Wael Mohamed, Sing-Yu Moorcraft, Basil Mary Eldo, Waqas Mehmood, Tawfik Elazzabi

**Affiliations:** Southwest Wales Cancer Centre, Swansea Bay University Health Board, Sketty Lane, SA2 8QA, Swansea, United Kingdom; Southwest Wales Cancer Centre, Swansea Bay University Health Board, Sketty Lane, SA2 8QA, Swansea, United Kingdom; Southwest Wales Cancer Centre, Swansea Bay University Health Board, Sketty Lane, SA2 8QA, Swansea, United Kingdom; Southwest Wales Cancer Centre, Swansea Bay University Health Board, Sketty Lane, SA2 8QA, Swansea, United Kingdom; Faculty of Community Medicine, Fatima Memorial Medical and Dental College, Shadman Rd, Ichhra, Lahore, 54000, Pakistan; Consultant Cellular Pathology, Swansea Bay University Health Board, Sketty Lane, SA2 8QA, Swansea, United Kingdom

**Keywords:** renal cell carcinoma, pancreatic metastasis, metachronous, stereotactic ablative radiotherapy

## Abstract

The pancreas is a rare site of distant metastasis from renal cell carcinoma. We present the case of a 68-year-old man with a prior history of left radical nephrectomy for renal cell carcinoma, who was found to have an isolated pancreatic metastasis in the pancreatic tail during routine follow-up imaging 4 years postsurgery. The patient was initially managed with pazopanib, but due to progressive growth of the pancreatic lesion without other metastatic sites, stereotactic ablative body radiotherapy (SABR) was given, with complete response at 12 months after SABR.

## Introduction

Pancreatic metastasis (PM) is a rare occurrence, typically seen in the absence of widespread systemic disease [1]. Among the primary tumours known to metastasize to the pancreas, renal cell carcinoma (RCC) is the most common, followed by malignancies such as lung, breast, and colorectal cancers [2]. PMs from RCC are typically identified in one of three scenarios: during the initial staging of the primary tumour, incidentally on routine surveillance imaging following treatment of the primary tumour, or when a patient presents with symptoms attributable to the pancreatic lesion, prompting further evaluation that reveals the underlying primary tumour [3, 4].

Surgery, in the form of pancreatectomy, is the primary treatment for most cases, depending on the patient’s performance status and underlying metastatic burden [5]. In a small number of cases, particularly when preserving pancreatic function is crucial, transarterial embolization (TAE), radiotherapy, and stereotactic ablative body radiotherapy (SABR) have been reported as alternative options [6].

This report presents a RCC case of isolated PM in the pancreatic tail, diagnosed 4 years after radical nephrectomy, which was successfully managed with pazopanib and SABR.

## Case report

**Figure 1:**
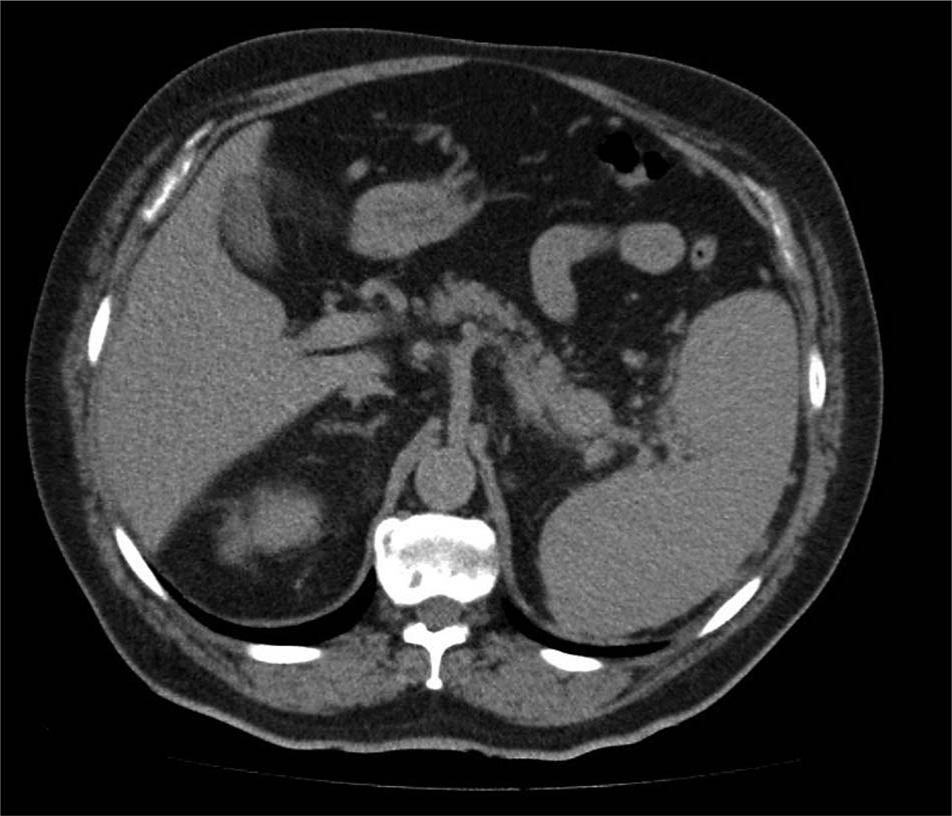
A contrast-enhanced CT scan of the abdomen (axial image) showing a soft tissue lesion in the pancreatic tail measuring 2.4 × 2.7 cm, radiologically suspicious for a primary pancreatic neoplasm.

A 68-year-old man with a prior history of left radical nephrectomy for RCC was incidentally found to have a pancreatic abnormality on surveillance imaging. A contrast-enhanced computed tomography (CT) scan of the abdomen showed a soft tissue lesion in the pancreatic tail measuring 2.4 × 2.7 cm, radiologically suspicious for a primary pancreatic neoplasm (Fig. 1). The left renal bed appeared clear.

**Figure 2:**
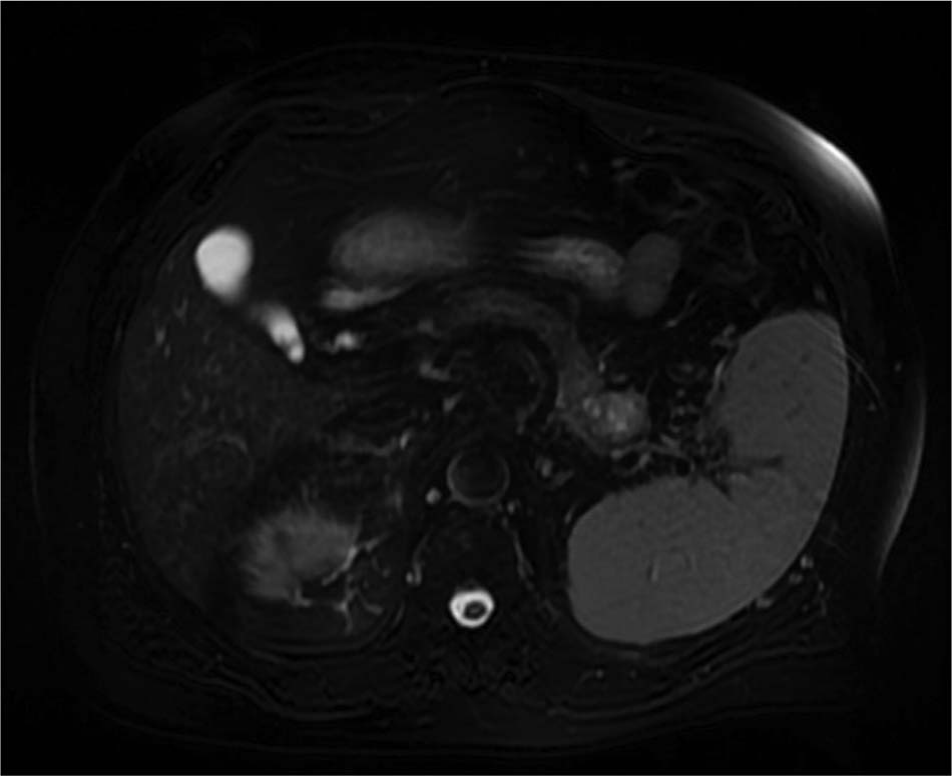
MRI (venous phase) shows a 2.6 cm lesion in the pancreatic tail, exhibiting heterogeneous *T*_2_ signal intensity with areas of high signal but lacking diffusion restriction.

Further characterization of the pancreas using MRI revealed a 2.6 cm lesion in the pancreatic tail, best visualized during the venous phase of the scan. The lesion exhibited heterogeneous *T*_2_ signal intensity with areas of high signal but lacked diffusion restriction (Fig. 2). Postcontrast images showed peripheral rim enhancement.

The patient had undergone left radical nephrectomy 4 years earlier for pT3aN0M0 clear cell RCC, Fuhrman grade 2. He had not received any adjuvant systemic therapy. His physical examination was unremarkable, and routine laboratory tests were within normal limits.

**Figure 3:**
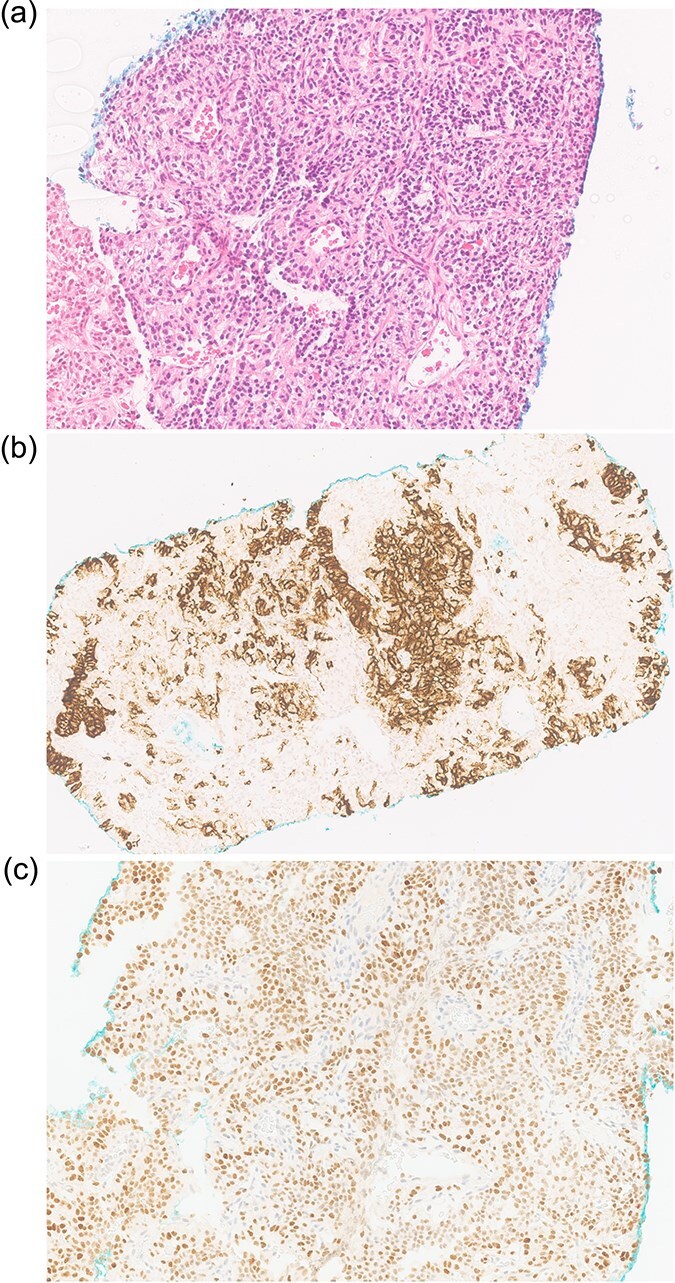
Biopsy shows (a) H&E scant cohesive clusters of polygonal epithelioid cells with clear cytoplasm. Immunohistochemistry positive for (b) CD10 and (c) PAX8, confirming metastatic RCC.

An ultrasound-guided biopsy revealed scant cohesive clusters of polygonal epithelioid cells with clear cytoplasm. Immunohistochemistry was positive for CD10 and PAX8, with weak expression of pancytokeratin (Fig. 3). The tumour cells were negative for synaptophysin, chromogranin, and cytokeratin 7, confirming the diagnosis of metastatic clear-cell RCC.

The patient was commenced on pazopanib 800 mg once daily. Follow-up imaging at 3 years demonstrated interval growth of the pancreatic lesion, with no evidence of metastases elsewhere. The patient received SABR to the PM with a total dose of 30 Gy in five fractions. Treatment planning adhered to established dose constraints for organs at risk (OARs), including maximum dose limits for the stomach (*D*_max_ < 30 Gy), duodenum (*D*_max_ < 32 Gy), small bowel (*D*_max_ < 30 Gy), liver (V15Gy < 700 cc), and spinal cord (*D*_max_ < 25 Gy). The treatment was well tolerated, with no reported acute toxicities.

At 12-month follow-up following SABR and ongoing pazopanib therapy, CT imaging signified a complete radiological response in the treated PM. No delayed side effects were noted.

## Discussion

RCC typically metastasises to the lungs, liver, lymph nodes, and brain [1]. PMs are rare, occurring in <2% of advanced RCC cases [2]. Isolated metachronous PM often reflects a low tumour aggressiveness, with a prolonged median interval of 7.1–10.0 years from initial RCC diagnosis to metastasis [7]. It is postulated that RCC micrometastases may persist within the tumour microenvironment (TME), gradually establishing in the pancreas by promoting angiogenesis and immune evasion through cytokine release [8].

Most PM patients remain asymptomatic, with lesions typically discovered incidentally during radiologic surveillance [6]. The delayed onset and nonspecific symptoms make early detection difficult. Imaging characteristics help differentiate PM from primary pancreatic tumours: RCC metastases are hypervascular during arterial phases on CT and MRI, unlike the hypovascular appearance of pancreatic adenocarcinoma [9]. However, differentiating them from pancreatic neuroendocrine tumours (pNETs) remains a challenge due to similar enhancement patterns. In such cases, CT-guided biopsy becomes essential for definitive diagnosis [10], as in our case.

Surgical resection offers a survival advantage in isolated PM from RCC, showing a 5-year survival rate of 75% and recurrence of ~40% [9]. SABR has emerged as a noninvasive, organ-sparing alternative to pancreaticoduodenectomy. In a pilot study, 11 patients with 31 PM cases were treated with MR-guided SABR. The authors reported 1-year and 2-year local control rates of 100% and 95%, respectively [11].

In another study, 20 PM in 16 RCC patients were managed with SABR. With a median follow-up of 35.9 months, the authors reported a local control rate of 90%. The median progression-free survival (PFS) was 12 months, and the 2-year overall survival (OS) was 100%. The most commonly used SABR regimen was 40 Gy in five fractions [12].

A recent study evaluated 51 RCC patients with PM, comparing outcomes following pancreaticoduodenectomy, SABR versus systemic therapy. With a median follow-up of 25 months, the median PFS was 36 months for surgery/SABR and 22 months for systemic therapy, though the difference was not statistically significant (*P* = .3). Similarly, OS outcomes were comparable, with a median OS of 121 months for surgical/SABR patients and not reached for those on systemic therapy (*P* = .52) [13].

SABR was selected for our patient due to several key factors: (i) a solitary, radiologically accessible PM with a favourable biology and no other disease sites; (ii) a good performance status and a preference to avoid surgery to achieve high local control with minimal toxicity; (iii) the size and location of PM, which allowed for safe delivery within OAR constraints; and (iv) a multidisciplinary approach.

## Conclusion

SABR provides an alternative option to surgery in treating isolated PM of RCC cases, particularly in patients with extended disease-free periods or those unsuitable for surgery. Given its efficacy and safety profile, SABR is a viable option in MDT boards as a curative modality of intent.
